# A simple validation and screening method for CRISPR/Cas9-mediated gene editing in mouse embryos to facilitate genetically modified mice production

**DOI:** 10.1371/journal.pone.0312722

**Published:** 2025-03-27

**Authors:** Dawid Winiarczyk, Hossein Khodadadi, Paweł Leszczyński, Hiroaki Taniguchi

**Affiliations:** 1 Department of Experimental Embryology, Institute of Genetics and Animal Biotechnology, Polish Academy of Sciences, Jastrzebiec, Poland; 2 Department of Stem Cell Bioengineering, Mossakowski Medical Research Institute, Polish Academy of Sciences, Warsaw, Poland; 3 African Genome Center, Mohammed VI Polytechnic University (UM6P), Ben Guerir, Morocco; Hirosaki University Graduate School of Medicine, JAPAN

## Abstract

Clustered regularly interspaced short palindromic repeats/CRISPR-associated protein 9 (CRISPR/Cas9) is a genome engineering method for generating site-specific editing in target genes in a variety of species. It is a common tool for generating mouse models of different diseases. However, detecting target modifications in mouse embryos can be time-consuming and expensive. Accordingly, developing a screening method to confirm gene modification may be useful. We propose herein an evaluation method (cleavage assay – CA) for CRISPR/Cas9-mediated gene editing in preimplantation mouse embryos that allows us to detect mutants efficiently and later on initiate *in vivo* production without the extensive number of samples needing to be sent for Sanger sequencing and animal usage. Our method is based on the inability of the RNP complex to recognize the target sequence after CRISPR-mediated genome editing due to modification of the target locus. It allows us to establish gene edited mice in a user-friendly fashion with a limited number of mice usage by confirming each step of CRISPR-mediated gene editing of mouse embryos and, therefore, can be considered as a supportive tool to existing procedures for verification of successful CRISPR/Cas9-mediated gene alterations in mouse embryos and further mutant production.

## Introduction

Genome engineering technologies have developed rapidly and generated a wide range of applications in many fields, such as biology, medicine, and even agriculture [1–4]. CRISPR/Cas9 is a bacterial immune system adopted by scientists as a simple and precise tool for gene editing. Due to the generation of double-stranded breaks (DSB) by the ribonucleoprotein complex (RNP) and the cellular pathways that repair them, we can easily induce modifications in a target gene to study its function [5,6]. One of the methods widely used to confirm the success of a target modification is the T7 endonuclease I (T7EI) assay [7,8]. However, the T7EI method needs extra reagents and steps to confirm the success of gene editing. Direct Sanger sequencing is also available to determine the success rate of genome editing in mammalian embryos [9]. Nevertheless, Sanger sequencing analysis is time-consuming, especially in a lab that relies on outsourcing services. Although these existing methods are well established and valuable, we propose an alternative method to evaluate gene-edited mouse embryos as the user’s choice, which helps the prediction of success in CRISPR-mediated gene editing before proceeding to embryo transfer to generate gene-edited mice in a small number of experimental samples. Our screening method is based on the inability of the RNP complex to recognize the target sequence after CRISPR-mediated genome editing due to modification of the target sequence. Moreover, with limited mouse usage, it allows us to establish gene edited mice in a user-friendly, cost and time-effective fashion. Additionally, using fluorescently labeled guide RNA (gRNA), our method allows us to determine the success of introducing the RNP complex into the embryos, which is important for the CRISPR-mediated gene editing process and could be useful for gene editing in the embryos of other animals.

## Materials and methods

The protocol described in this peer-reviewed article is published on protocols.io.

### Animals

All animal experiments were carried out in the Institute of Genetics and Animal Biotechnology of the Polish Academy of Sciences (IGAB PAS). Mature (C57BL/6 ×  CBA/H) F_1_ mice, approximately 3 months old and originating from the IGAB PAS colony, were used in this study. They were kept in a temperature-controlled room with a 12 h light:12 h darkness cycle (lights on from 06:00 to 18:00 h). Food (Labofeed H, Kcynia, Poland; metabolic energy of 13.0 MJ/kg) and water were available *ad libitum*. The experiments were performed according to the recommendations of the Polish Governmental Act for Animal Care. They were approved by the II Local Ethical Committee for Experiments on Animals in Warsaw (Approval no. WAW2/192/2018).

### Mouse zygote preparation

For zygote collection, donor F_1_ females were superovulated by intraperitoneal injection of 7.5 IU of pregnant mare’s serum gonadotropin (PMSG) (Folligon, Intervet, Netherlands), followed by 7.5 IU of human chorionic gonadotropin (hCG) (Chorulon, Intervet, Netherlands) 48–52 h later. They were mated with F_1_ males immediately after the hCG injection. Zygotes were collected from the oviducts of donor females sacrificed by cervical dislocation 20–24 h after the hCG injection. The oviducts were collected and placed in a hyaluronidase solution (150 IU/ml M2). Zygotes surrounded by follicular cells were released from the ampulla of the oviduct. After approximately 5 minutes, the follicular cells were dispersed, and the zygotes were collected and washed 3 times in the M2 medium.

### Production of gRNA and RNP complex

For the zygote electroporation, guide RNA (gRNA) was prepared in a 0.5 ml tube by mixing 0.6 µl (100 µM) crRNA and 0.6 µl (100 µM) tracrRNA each with 1.8 µl Nuclease-Free Duplex Buffer (Cat. #11-01-03-01; IDT, Coralville, USA). The reaction was incubated at 95°C for 3 min and cooled down for 30 min after turning off the heatblock to generate annealed gRNA. 0.96 µl of NLS-Cas9 (IDT; 61 µM) and 6.04 µl of Opti-MEM I (Life Technologies, Carlsbad, California, U.S.) were then added (Total 10 µl). For the cleavage assay, guide RNA (gRNA) was prepared in a 0.5 ml tube by mixing 0.5 µl (100 µM) crRNA and 0.5 µl (100 µM) tracrRNA each with 49 µl Nuclease-Free Duplex Buffer and the mixture was incubated at 95°C for 3 min and cooled down for 30 min after turning off the heat-block to generate annealed gRNA. 61 µM NLS-Cas9 (IDT) was diluted in Opti-MEM I to 1 µM concentration. Hprt1 crRNA sequence: ACCTCTTAGGAGTCTAAAGT. Mecom crRNA sequence: TCTCTAACCTTTGCAGATCG.

### Electroporation

The Genome Editor electroporator and LF501PT1-10 platinum plate electrode (length: 10 mm, width: 3 mm, height: 0.5 mm, gap: 1 mm) (BEX Co. Ltd., Tokyo, Japan) were used for electroporation. The electrode was connected to the electroporator and was set under a stereoscopic microscope. The prepared zygotes in the RNP complex solution were subjected to electroporation (up to 40 zygotes at a time). Zygotes were washed with Opti-MEM I 3 times to remove the serum in the medium, placed in a line in the electrode gap filled with 5 µl of Opti-MEM I containing a mixture of 0.48 µl of 61 µM Cas9 protein and 0.3 µl of annealed gRNA (100 µM crRNA and tracrRNA), and subjected to electroporation. The electroporation conditions were set to 30 V (3 ms ON + 97 ms OFF) 10 pulses in most experiments [10,11]. After the electroporation, the zygotes were immediately collected from the electrode chamber and subjected to 4 washes with M2 medium followed by 3 washes with KSOM medium. The embryos were then cultured in KSOM medium at 37°C and 5% CO_2_ in an incubator until the blastocyst stage.

### Embryo culture

The embryos were cultured *in vitro* in drops (15 embryos per 20 µL) of KCl-enriched simplex optimized medium (KSOM) (Specialty Media, Phillipsburg, NJ, USA), supplemented with amino acids and 4 mg/ml BSA, under paraffin oil in an atmosphere of 5% CO_2_ in air at 37.5°C.

### Vasectomy

Two-month-old F1 males were anesthetized with intraperitoneal injections of xylazine and ketamine (10–16 and 80–120 mg/kg, respectively). After shaving the abdomen of each animal and cleaning it with alcohol, an incision was made to access the body cavity and expose the vas deferens. The vas deferens were cut and tied off with sutures, the body cavity was sewn up, and the mouse was transferred to a warm and clean cage until it recovered. Prior to being used for experiments, the sterility of vasectomized males was checked at 20 and 30 days post-surgery.

### Embryo transfer

Oviduct embryo transfer was performed 1h after electroporation to 3-4-month-old pseudopregnant F1 females mated with proven vasectomized males. Recipient female mice were intraperitoneally anesthetized with xylazine and ketamine (10–16 and 80–120 mg/kg, respectively). After cleaning with alcohol and shaving the back of the animal, a small incision was made. The ovary, oviduct, and part of the uterine horn were exposed and immobilized with a clamp holding the ovarian fat pad. The fallopian funnel of the oviduct was exposed, and a thin, fire-polished glass pipette (diameter ~ 80 µm) was used to transfer 10 zygotes in one oviduct. After embryo transfer, females were kept in their cages on a warm plate at 37°C overnight (O/N), then transferred to the colony and left undisturbed until delivery.

### Confocal microscopy

The localization of the RNP complex (NLS Cas9 and gRNA complex) was visualized with a Nikon A1R confocal microscope (Tokyo, Japan) 24h after electroporation. The images were captured using the NIS-Elements package. Confocal images were analyzed using IMARIS 6.0.1 software (Bitplane AG, UK).

### DNA extraction from mouse blastocysts

Blastocyst stage embryos were separately transferred using a mouth pipette to the 0.2 mL PCR tube and resuspended in 20 µl of lysis buffer, which consists of 100 mM Tris-HCl (pH =  8.3), 100 mM KCl, 0.45% Tween 20, 0.02% gelatin, and 125 µg/mL proteinase K. Lysis was performed by incubation at 56 °C for 10 min followed by 10 min denaturation at 95 °C. Crude lysate was used for direct DNA amplification or stored at -20 °C.

### PCR and agarose gel electroporation

The polymerase chain reaction (PCR) was performed using Terra™ PCR Direct Polymerase Mix (Cat. #639287; Takara Bio, Inc., Shiga, Japan) consisting of 12.5 µl of 2X Terra PCR Direct Buffer (with Mg^2+ ^, dNTP), 0.5 µl (1.25 U) Terra PCR Direct Polymerase Mix with the addition of 1 µl (10 µM) forward (F) and reverse (R) primer each, 1.5 µl of DNA template and sterile water up to final volume of 25 µl. PCR conditions were: the initial denaturation step of 98°C for 2 min, followed by 36 cycles of 98°C for 10 sec, 62°C for 15 sec, 68°C for 1 min, and a final extension of 68°C for 5 min. After completed amplification, 3 µl of PCR product was loaded onto 1.5% agarose gel containing 1 X TAE (Tris-acetate -EDTA) buffer (Cat. #15558042; Invitrogen™, Waltham, USA) and stained with 5 µl of SimplySafe™ per 100 ml of agarose solution (Cat. #E4600-01; EURx Ltd, Gdańsk, Poland). Electrophoresis was performed at 100 V for 30 minutes with a ladder for precise sizing.

Hprt1 primers:
  Fprimer: AGGTTTCGAGCCCTGATATTCG
  Rprimer: ATGTGGCAAGGTCAAAAACAGT
Mecom primers:
  Fprimer: GGCCAGTTGTTTTGAAGCTC
  Rprimer: GGACTTTTGGATCCCACCTT

### Cleavage assay

1µl of gRNA and 1µl of diluted NLS-Cas9 (1 µM in Opti-MEM I) and 2.5 µl of PCR products, 15.5 µl of Opti-MEM I were mixed and incubated for O/N. Electrophoresis was performed at 100 V for 30 minutes with a ladder for precise sizing, and the image was taken by ChemiDoc XRS + System (Bio-Rad) and Image Lab Software.

### Sanger sequencing

PCR products were sequenced using the primers by Sanger sequencing (Genomed S. A., Warsaw, Poland). The results were analyzed using FinchTV 1.4.0 software.

## Expected results and discussion

**Fig 1:**
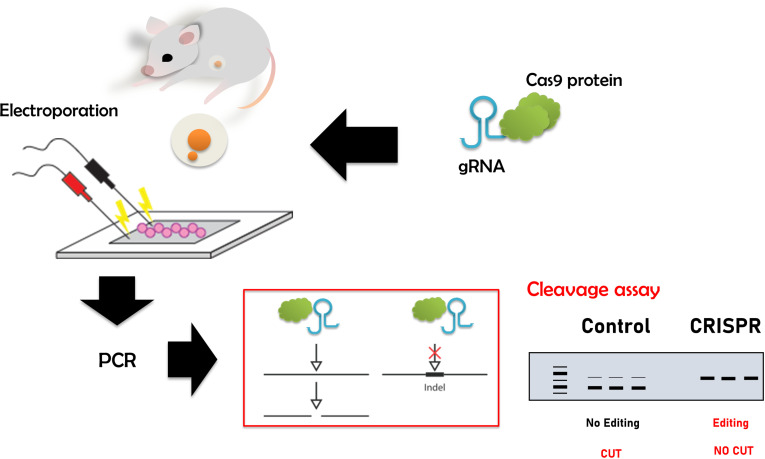
Workflow of a simplified method to detect successful CRISPR-mediated gene editing. After electroporating the RNP complex into the embryos, DNA is collected for PCR reaction. A cleavage assay confirms the success of CRISPR. When CRISPR successfully introduces deletions and insertions, the amplified PCR product is not cleaved by the Cas9 enzyme in complex with the gRNA used for CRISPR-mediated gene editing.

**Fig 2:**
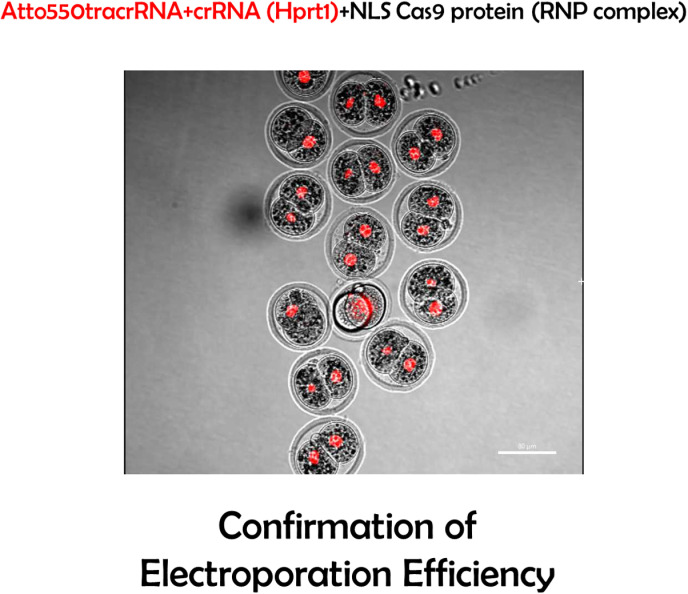
Fluorescence images of the nuclear-localized *Hprt1* specific gRNA- NLS-Cas9 RNP complex. ATTO550 signal is visualized in the nucleus 24h post electroporation.

**Fig 3:**
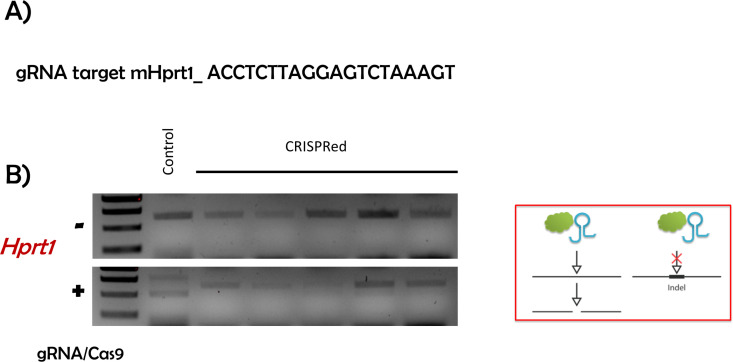
Validation of Hprt1 gRNA. A) gRNA target sequence and B) gel image of an in vitro cleavage assay to determine the efficacy of CRISPR/Cas9-mediated gene editing. When the cleavage is efficiently executed, the control PCR product (m*Hprt1* gene; 647 bp) is cleaved into approximately 509 and 138 bp products (a smaller size of cleavage product 138 bp was not seen clearly in the gel due to the primer dimers in our experimental condition, but 509 bp can assure the fact of the cleavage by gRNA). DNA ladder size is indicated at 750, 500, and 250 bp.

**Fig 4:**
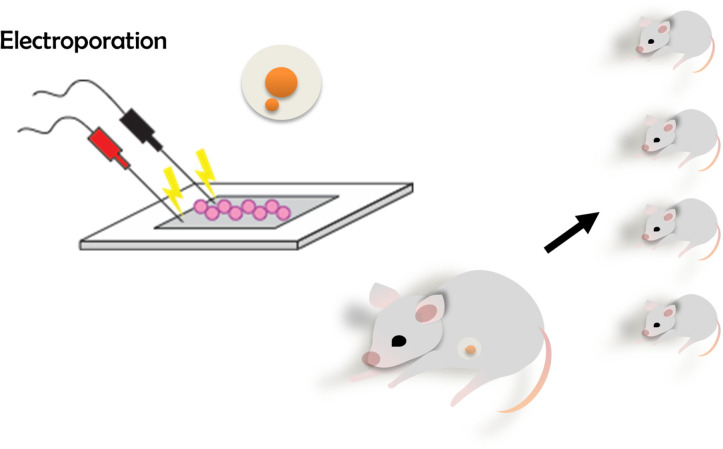
After electroporating the RNP complex into the embryos, the embryos are transferred to the foster mother to generate gene edited mice.

**Fig 5:**
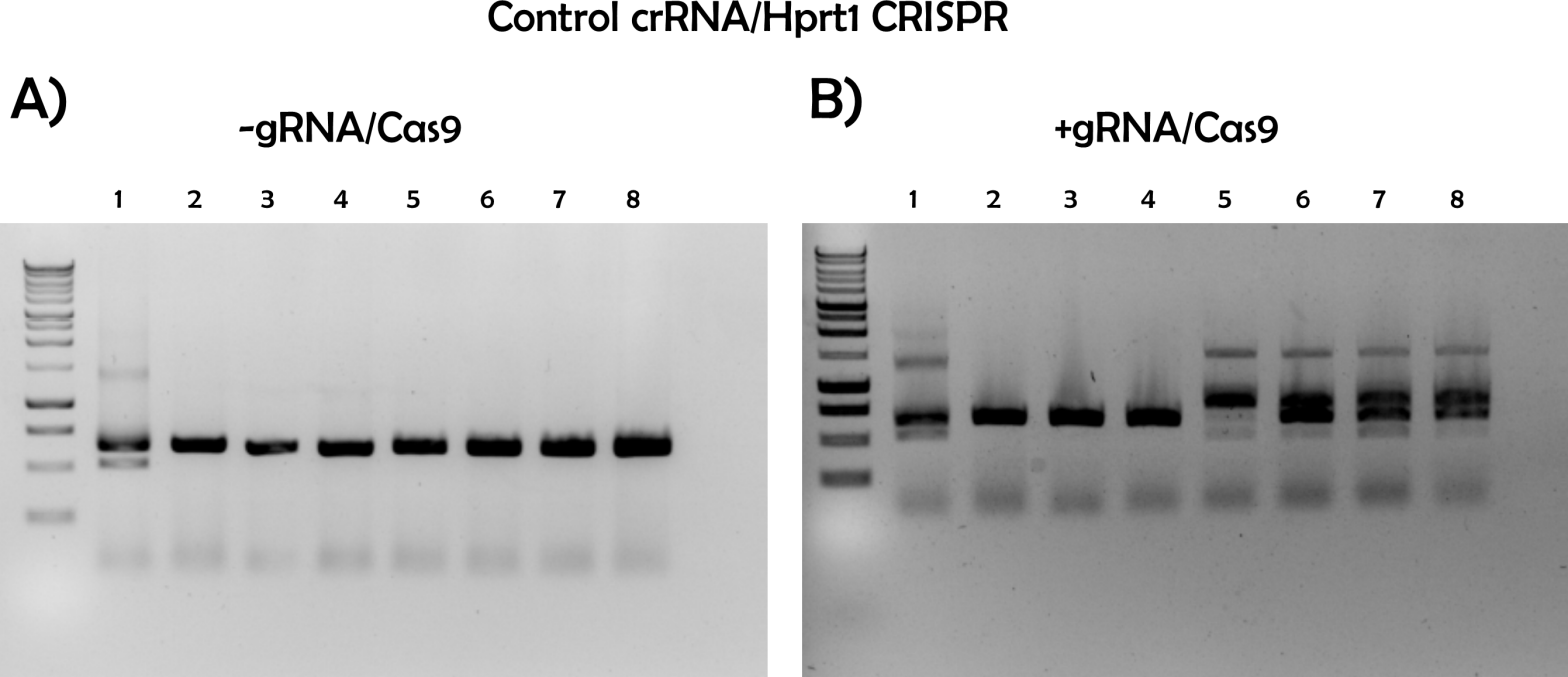
Validation of CRISPR-mediated gene editing of the *Hprt1* gene in postnatal mice generated *via* embryo transfer of gene edited embryos. Gel image of A) original PCR products and B) PCR products after *in vitro* cleavage assay to determine the efficacy of CRISPR/Cas9-mediated gene editing.

**Fig 6:**
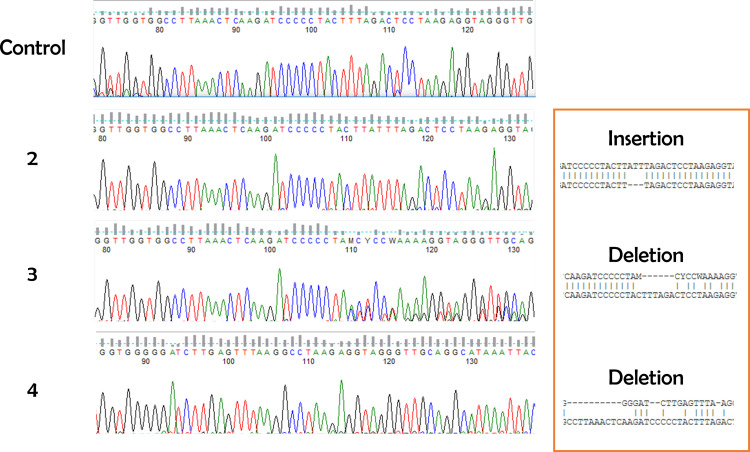
Direct PCR-based Sanger sequencing analysis of the *Hprt1* gene locus with CRISPR modification in postnatal mice.

CRISPR/Cas9-mediated gene editing has revolutionized many different fields [12]. We present an approach that is both time- and cost-efficient in evaluating the result of zygote electroporation combined with cloning-free CRISPR (Fig 1). To validate the accuracy of the designed crRNA, we targeted the *Hprt1* gene, which is commonly used (Fig 3A and B). Next, we electroporated the RNP complex consisting of Hprt1 crRNA, ATTO550 tracrRNA, and NLS-Cas9 protein to the mouse zygote. After 24h, as shown in Fig 2, the red fluorescent (ATTO 550) labeled RNP complex is primarily located in the nucleus of mouse embryos. This confirms that post-electroporation, the RNP complex translocates to the nucleus (due to NLS combined with Cas9 protein) to edit the target locus without affecting the viability of the embryos (S1 and S2 Figs). This method allows us to visualize RNP complex localization in the nucleus and, together with data from the cleavage assay, shown in Fig 3A and B, enables us to predict the success of CRISPR-mediated gene editing. In the cleavage assay, original PCR bands remain when the editing occurs in both alleles (homozygote), and when the editing does not occur or happens in one allele (heterozygote), at least fragments are visible on the gel (Figs 3B and S3). In the control blastocyst, there is neither deletion nor insertion. Therefore, while the targeting RNP complex normally cuts it, the edited DNA of the blastocyst is not recognized by the complex due to deletion or insertion of a few nucleotides caused by CRISPR-mediated editing (Figs 3B, S3). Therefore, our approach provides a dual confirmation of the success of CRISPR-mediated gene editing by visualizing RNP complex in the embryos and performing CA. Importantly, in our CA method, we can validate CRISPR-mediated editing at very user-friendly conditions using very simple commercially available chemicals such as Cas9 protein, gRNA, and PCR products from the target gene locus, so no additional reagents are required. Consistent with the confirmation of the success of CRISPR-mediated editing using our CA method, our newly prepared gene edited embryo successfully developed in postnatal mice with gene modification in the target locus (Fig 4-6). Cleavage assay (Fig 5) and Sanger sequencing (Fig 6) support the idea that we could introduce homozygote mutation in the target gene element as indicated.

Although our approach is straightforward and cost-effective, it may not detect mutations involving single-nucleotide insertions or deletions. However, in most cases, CRISPR/Cas9-mediated gene modification is associated with deletions and insertions of more than one nucleotide. Similar to our approach, the commonly used T7EI-based method cannot also detect single nucleotide nicks [13]. Therefore, our established method can detect the CRISPR-mediated gene edition at the same level as T7EI-based detection and supports the usefulness of our developed method. Moreover, our protocol is user-friendly (basic embryological and molecular skills are needed). Although existing detection methods such as T7EI and Sanger methods are quite established and practical, our method can be considered an alternative method as a user’s choice and would be helpful from an animal ethics point of view as it may reduce the animal number in experiments and breeding cost. Our experiment did not detect the effect of ATTO550 on embryo development. However, one can use this marker to determine the efficiency of electroporation and use nonlabelled tracrRNA for animal development if embryo production is affected by it for unknown reasons. Nevertheless, it is assumed that our method can detect the efficiency of RNP complex introduction into the embryos and is, therefore, useful to optimize the electroporation condition for many different animal embryos and of potential use for genetic engineering in agriculture. Further studies are needed to prove this point. Additionally, targeting an exon using two sgRNAs (or crRNAs) instead of introducing an indel could be a useful strategy for gene knockout.

## Supporting information

The protocol described in this peer-reviewed article is published on protocols.io, https://dx.doi.org/10.17504/protocols.io.36wgqd91yvk5/v1.

## Supporting information

S1 Fig Localization of RNP complex and Atto550 tracrRNA in the 2 cell stage of the embryos. Hprt1 RNP complex and only Atto550 tracrRNA were electroporated and observed after 24h. Only the Hprt1 RNP complex is located in the nucleus of the embryos at the 2 cell stage, scale bar 50 um. A) confocal red channel for Atto550 tracrRNA detection; B) bright field; C) merge.
(TIF)

S2 Fig Atto550 tracrRNA does not affect the development of the embryos. Localization of RNP complex and Atto550 tracrRNA at the 1 cell (4h) and 2 cell stage (24h) of the embryos.
(TIF)

S3 Fig Mecom Exon 4 is targeted by specific RNP complexes in the mice embryos. Gel image of an in vitro cleavage assay to determine the efficacy of CRISPR/Cas9-mediated gene editing. When the cleavage is efficiently executed, the Mecom Exon4 PCR product (375 bp) from the control embryo is cleaved into approximately 260 and 115 bp products. DNA ladder size is indicated at 500 and 250 bp, respectively. Sequencing results show deletion and insertion in samples 4 and 6.
(TIF)

## References

[1] Adli M. The CRISPR tool kit for genome editing and beyond. Nat Commun. 2018;9:1911. 29765029 10.1038/s41467-018-04252-2 PMC5953931

[2] Hsu PD, Lander ES, Zhang F. Development and applications of CRISPR-Cas9 for genome engineering. Cell. 2014;157(6):1262–78. doi: 10.1016/j.cell.2014.05.010 24906146 PMC4343198

[3] Manghwar H, Lindsey K, Zhang X, Jin S. CRISPR/Cas System: Recent Advances and Future Prospects for Genome Editing. Trends Plant Sci. 2019;24(12):1102–25. doi: 10.1016/j.tplants.2019.09.006 31727474

[4] Xiao-Jie L, Hui-Ying X, Zun-Ping K, Jin-Lian C, Li-Juan J. CRISPR-Cas9: a new and promising player in gene therapy. J Med Genet. 2015;52(5):289–96. doi: 10.1136/jmedgenet-2014-102968 25713109

[5] Chu HW, Rios C, Huang C, Wesolowska-Andersen A, Burchard EG, O’Connor BP, et al. CRISPR-Cas9-mediated gene knockout in primary human airway epithelial cells reveals a proinflammatory role for MUC18. Gene Ther. 2015;22(10):822–9. doi: 10.1038/gt.2015.53 26043872 PMC4600011

[6] Takeda H, Kataoka S, Nakayama M, Ali MAE, Oshima H, Yamamoto D, et al. CRISPR-Cas9-mediated gene knockout in intestinal tumor organoids provides functional validation for colorectal cancer driver genes. Proc Natl Acad Sci U S A. 2019;116(31):15635–44. doi: 10.1073/pnas.1904714116 31300537 PMC6681705

[7] Kim JM, Kim D, Kim S, Kim J-S. Genotyping with CRISPR-Cas-derived RNA-guided endonucleases. Nat Commun. 2014;5:3157. doi: 10.1038/ncomms4157 24445736

[8] Vouillot L, Thélie A, Pollet N. Comparison of T7E1 and surveyor mismatch cleavage assays to detect mutations triggered by engineered nucleases. G3 (Bethesda). 2015;5(3):407–15. doi: 10.1534/g3.114.015834 25566793 PMC4349094

[9] Sakurai T, Watanabe S, Kamiyoshi A, Sato M, Shindo T. A single blastocyst assay optimized for detecting CRISPR/Cas9 system-induced indel mutations in mice. BMC Biotechnol. 2014;14:69. doi: 10.1186/1472-6750-14-69 25042988 PMC4118159

[10] Hashimoto M, Takemoto T. Electroporation enables the efficient mRNA delivery into the mouse zygotes and facilitates CRISPR/Cas9-based genome editing. Sci Rep. 2015;5:11315. 10.1038/srep11315 PMC4463957 26066060

[11] Hashimoto M, Yamashita Y, Takemoto T. Electroporation of Cas9 protein/sgRNA into early pronuclear zygotes generates non-mosaic mutants in the mouse. Dev Biol. 2016;418(1):1–9. doi: 10.1016/j.ydbio.2016.07.017 27474397

[12] Pickar-Oliver A, Gersbach CA. The next generation of CRISPR-Cas technologies and applications. Nat Rev Mol Cell Biol. 2019;20(8):490–507. doi: 10.1038/s41580-019-0131-5 31147612 PMC7079207

[13] Guan C, Kumar S, Kucera R, Ewel A. Changing the enzymatic activity of T7 endonuclease by mutations at the beta-bridge site: alteration of substrate specificity profile and metal ion requirements by mutation distant from the catalytic domain. Biochemistry. 2004;43(14):4313–22. doi: 10.1021/bi036033j 15065875

