# Supplementary figures and images for: A simple validation and screening method for CRISPR/Cas9-mediated gene editing in mouse embryos to facilitate genetically modified mice production
Source: PLoS One. 2025 Mar 27;20(3):e0312722. doi: 10.1371/journal.pone.0312722 (PMC11949363; doi:10.1371/journal.pone.0312722)

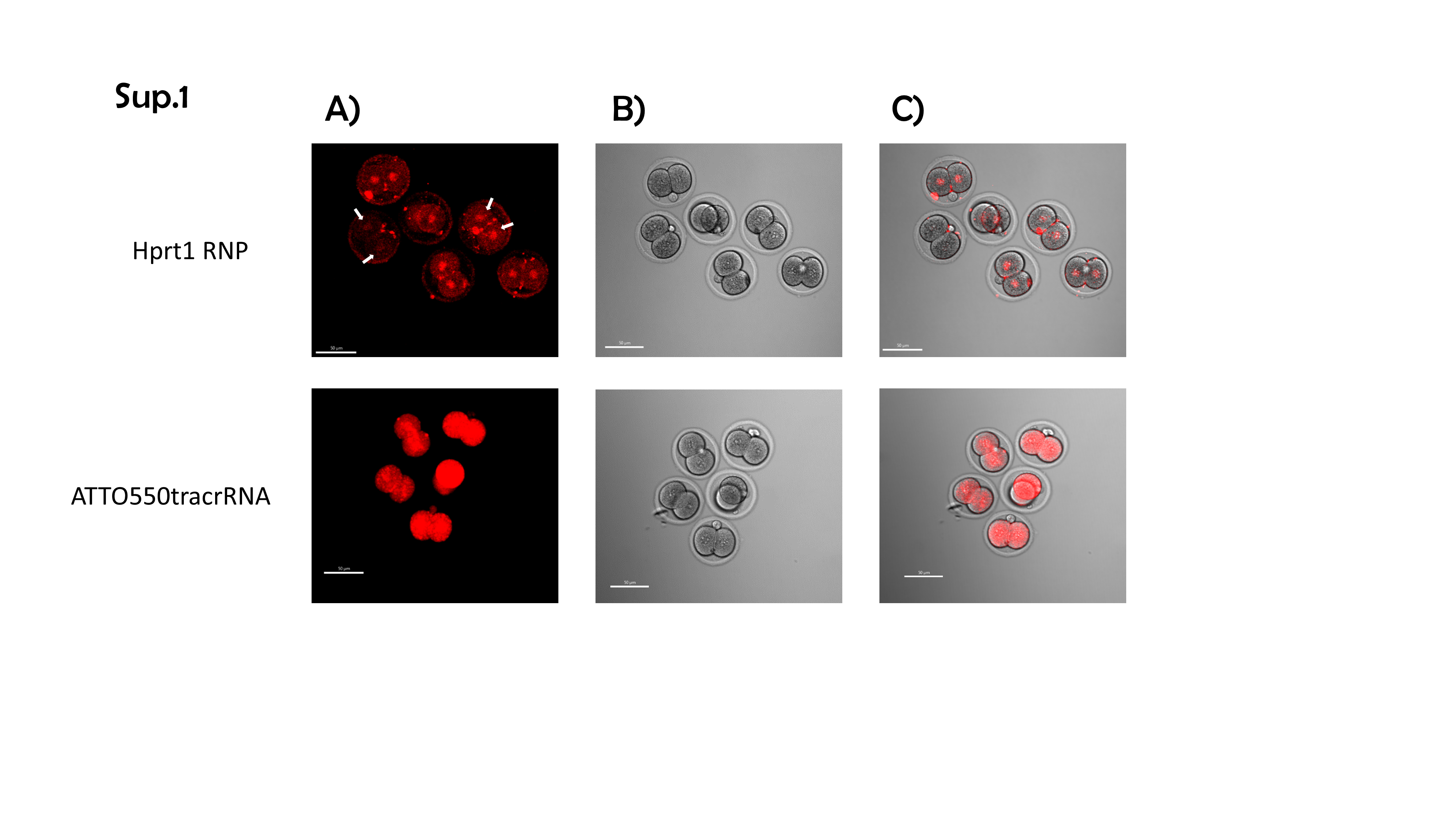

Supplement: S1 Fig — Hprt1 RNP complex and only Atto550 tracrRNA were electroporated and observed after 24h. Only the Hprt1 RNP complex is located in the nucleus of the embryos at the 2 cell stage, scale bar 50 um. A) confocal red channel for Atto550 tracrRNA detection; B) bright field; C) merge. (TIF) [file pone.0312722.s001.TIF]

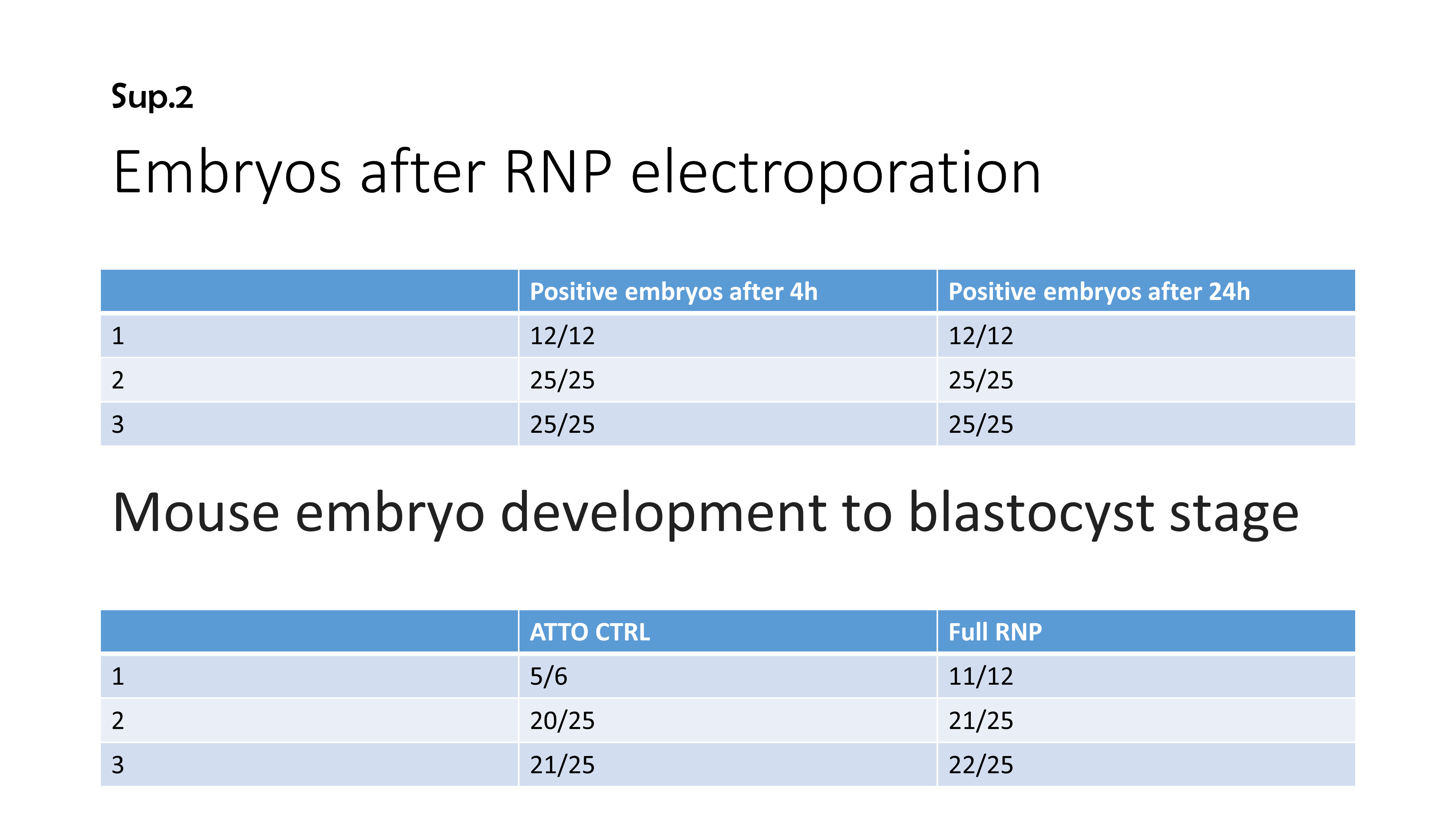

Supplement: S2 Fig — Localization of RNP complex and Atto550 tracrRNA at the 1 cell (4h) and 2 cell stage (24h) of the embryos. (TIF) [file pone.0312722.s002.TIF]

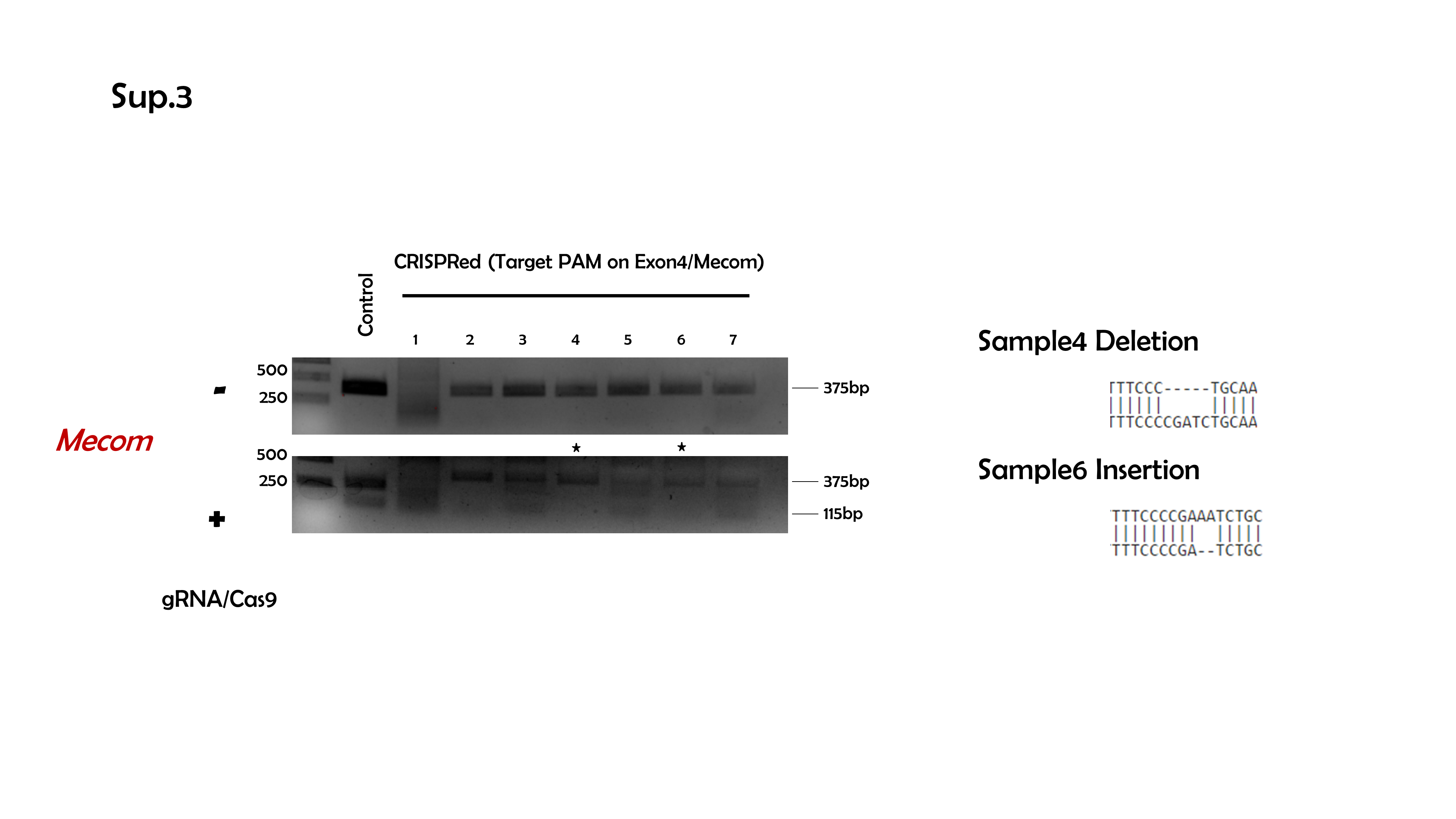

Supplement: S3 Fig — Gel image of an in vitro cleavage assay to determine the efficacy of CRISPR/Cas9-mediated gene editing. When the cleavage is efficiently executed, the Mecom Exon4 PCR product (375 bp) from the control embryo is cleaved into approximately 260 and 115 bp products. DNA ladder size is indicated at 500 and 250 bp, respectively. Sequencing results show deletion and insertion in samples 4 and 6. (TIF) [file pone.0312722.s003.TIF]
